# Cardiovascular Consequences of Repetitive Arousals over the Entire Sleep Duration

**DOI:** 10.1155/2017/4213861

**Published:** 2017-09-05

**Authors:** Yanyan Wu, Rong Huang, Xu Zhong, Yi Xiao

**Affiliations:** Department of Respiratory Medicine, Peking Union Medical College Hospital, Chinese Academy of Medical Sciences and Peking Union Medical College, Beijing, China

## Abstract

**Objectives:**

To explore the cardiovascular effects of nightlong repetitive arousals (RA).

**Methods:**

Twenty healthy subjects participated in two consecutive sleep studies. The first one was free of intervention and the second study involved repetitive arousals induced by acoustical stimuli. Blood pressure, heart rate variability (HRV), arterial stiffness index (ASI), and serum markers including nitric oxide (NO), endothelin-1 (ET-1), C-reactive protein (CRP), interleukin-6 (IL-6), tumor necrosis factor-*α* (TNF-*α*), and vascular endothelial growth factor (VEGF) were studied.

**Results:**

RA led to overnight elevation in diastolic blood pressure (DBP) but not in systolic blood pressure (SBP). Regarding HRV, overnight increase in low frequency power (LF) and low frequency to high frequency ratio (LHR) and decrease in high frequency power (HF) were evident. The relative overnight differences in HF and LHR correlated with the amount of rapid-eye movement (REM) sleep. RA did not cause detectable change in either ASI or serum markers of interest.

**Conclusions:**

Nightlong RA alters the sympathovagal modulation significantly and this effect seems to be associated with the amount of REM sleep. Exposure to RA also causes an elevation in postsleep DBP. Disturbance to autonomic nervous system (ANS) may precede endothelial dysfunction and increased arterial stiffness as cardiovascular consequences of RA.

## 1. Introduction

Obstructive sleep apnea (OSA) has been identified as an independent risk factor for cardiovascular diseases [1], but the mechanism involved has not been fully elucidated yet.

There are three basic pathophysiologic changes accompanying frequent apneic events in OSA patients, intermittent hypoxia, repetitive arousals, and large fluctuation of intrathoracic pressure [2]. It is uncertain to what extent and through which pathway that each of them contributes to the unfavorable cardiovascular outcome. The present study examined the effect of repetitive arousals on cardiovascular system independent of the other two factors.

Increased sympathetic nervous system activity is thought to be contributing to overall cardiovascular mortality in OSA patients [3]. In OSA, periodic episodes of increased heart rate and blood pressure caused by arousals have been described and are thought to be mediated by sympathovagal modulation [4]. It is hypothesized that the excitatory effect of repetitive arousals on the sympathetic nervous system (SNS) may be cumulative [5, 6], However, a recent study demonstrated that repetitive arousals lasting for an hour only resulted in subtle cumulative effect on the ANS [7]. In the present study, repetitive arousals were elicited over a much longer period to model the reality better and investigate whether the effects would become more evident.

Arousal can cause transient elevation of blood pressure [4, 8] and repetitive arousals attenuate the blood pressure dipping at night [9]. The present study also evaluated whether repetitive arousals can cause a more persistent change in blood pressure.

Endothelial dysfunction is another key factor mediating damage to cardiovascular system in OSA [10]. Repetitive arousals may impair the normal function of endothelium through several pathways [11–13]. The present study intended to evaluate the effect of repetitive arousals on endothelium in terms of its intrinsic vasoactive capability and inflammatory status. The markers used in this study include NO, ET-1, CRP, IL-6, TNF-*α*, and VEGF, all of which have been linked to both OSA and endothelial dysfunction [11, 12, 14, 15].

Increased arterial stiffness is strongly associated with atherosclerosis [16, 17] and has been reported in OSA patients, even with only mild disease [18, 19]. The current study used contour analysis of digital volume pulse (DVP) [20, 21] to investigate whether repetitive arousals lead to increase in arterial stiffness.

Repetitive arousals are an important component in OSA and its adverse cardiovascular consequences. The objective of the research was to explore whether repetitive arousal could independently lead to changes in the cardiovascular system, with particular reference to autonomic control status, blood pressure, endothelial function, and arterial stiffness.

## 2. Methods

### 2.1. Subjects

Twenty male subjects were recruited, with a mean age of 25.0 (2.1) y and a mean body mass index of 22.9 (1.9) kg·m^−2^. Questionnaire screening was conducted via telephone interview for history of cardiovascular or respiratory diseases, concomitant nervous system diseases, personal or family history of sleep disorders, typical symptoms of OSA, disordered wake-sleep rhythm, intensive consumption of caffeine or alcohol, travels crossing time zones in the past three months, major life stress events in the past three months, and hearing impairment. None of the participants was on long-term medication of any kind.

Subjects were asked to keep a sleep diary to record their sleep status and relevant events for a week immediately prior to the planned sleep studies. They were required to refrain from caffeine or alcohol consumption, any medication which would disturb sleep, and intensive physical exercise on the day. They were also asked to have a light dinner prior to sleep study.

This research was approved by the Ethics Review Committee of Peking Union Medical College Hospital, Chinese Academy of Medical Sciences. All subjects gave written informed consent before participation.

### 2.2. Experiment Design and Procedure

Each subject spent two consecutive nights in the sleep laboratory. The first night was free of any intervention. On the second night, auditory stimuli were applied repetitively throughout the entire sleep duration to induce arousals.

The procedure of presenting auditory stimuli was adapted from preliminary studies and the research done by Chaicharn et al. [7]. To minimize the possibility of completely waking up the subjects, the procedure started with a low intensity of stimulus and was advanced with small increments. The procedure contained a series of alterations in different features of sound, including intensity, frequency, duration, and repeat pattern. This diversity also helped to slow the auditory habituation.

After about five-minute stable stage 2 sleep, a tone of 1000 Hz with the intensity of 50 dB and the duration of 0.5 seconds was presented and a judgment about whether an arousal occurred was made by the experimenter immediately according to the American Academy of Sleep Medicine arousal scoring rule [22]. In the present study, if the shift of electroencephalogram (EEG) frequency lasts for more than 15 seconds, the term “wakefulness” was used instead of “arousal” because a 30-second epoch should be scored as “wakefulness” if it consisted of more than 50% alpha rhythm.

**Figure 1:**
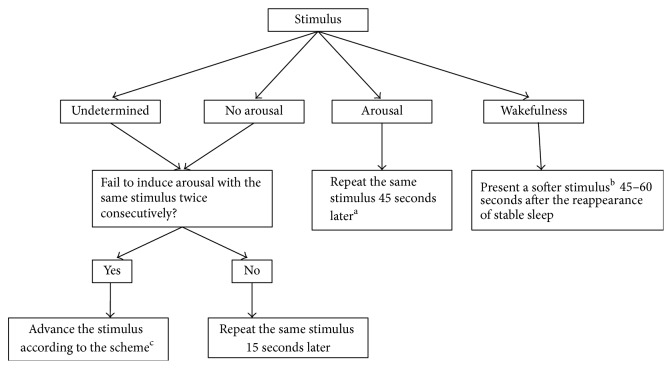
The modulation of stimuli based on the consequence of the previous stimulus. Arousal and wakefulness were defined in accordance with the AASM scoring rule. ^a^The stimuli were presented roughly every 45 seconds in order to achieve the expected arousal index, because not every stimulus would evoke arousal successfully. ^b^The set of stimulus presented before the current one was considered to be the softer one. ^c^The strategy to advance stimuli is illustrated in detail in the text.

If an arousal did not occur, stimulus duration was increased to one second and further increased to two seconds and then three seconds. If no arousal occurred, the stimulus intensities were increased by an increment of 5 dB to a maximal level of 80 dB. At each level of sound intensity, the stimulus duration increased from half second to three seconds gradually. If no arousal was induced with a stimulus of 80 dB presenting for three seconds, the tone was switched to a loop pattern with 0.5 seconds on and 0.5 seconds off repetitively (one-second loop). The loop stimulus could be paused immediately by the experimenter whenever an arousal was observed. The repeat pattern of the tones could be further advanced to 0.5-second, 0.2-second, and at the most 0.1-second loop to increase the efficacy of inducing arousals. In case habituation began to appear, frequency of the tones was increased by an increment of 200 Hz to a maximum of 2200 Hz while other aspects of the tones remained the same. Spontaneous arousals also accounted for total arousal index (ArI), and if one occurred, stimulus was not presented until 45 seconds after the subject fell back to sleep.  Figure 1 described how the stimuli were adjusted based on the instantaneous judgment.

The tones were generated and adjusted by Test Tone Generator (Version 4.32, Personal License, Timo Esser, Germany) and administrated with Edifier Super Woofer M3 speaker system (Edifier® Technology Co., Ltd., Beijing, China). The subwoofer was positioned 20 cm vertically above participants' heads with the two satellites placed 50 cm apart and 60 cm above the head position.

### 2.3. Polysomnography

The time of lights out was primarily determined by the routine sleep habit of the individual subject and the time of lights on in the morning was estimated with the time of falling asleep on the previous night and the usual sleep duration as reported in sleep diary. Participants were required to stay awake undisturbedly in supine position for ten minutes before lights out and after lights on, respectively.

The polysomnography recordings were conducted by Embla N7000 Recording System (Embla Systems Inc., Colorado, USA) and included four EEG leads (C3-M2, C4-M1, O1-M2, and O2-M1), two electrooculogram leads, chin electromyogram, single-channel of electrocardiogram (ECG, sampling rate 200 Hz), respiratory inductance pattern from thoracic and abdominal plethysmography, nasal flow, snoring sound, and pulse oximetry placed at the index finger of the left hand (sampling rate 100 Hz).

Sleep study results were scored automatically with manual correction and reviewed by experienced sleep medicine specialist.

### 2.4. Signal Processing and Measurements

#### 2.4.1. Heart Rate Variability Analysis

A five-minute ECG segment with minimal noise interference was selected from the data recorded over the 10-minute supine rest. The data was exported and analyzed with the Kubios HRV toolkit (Kubios HRV, version 2.1, Biosignal Analysis and Medical Imaging Group, Kuopio, Finland). R waves were automatically detected and reviewed manually. The HRV variables were calculated via fast Fourier transform (FFT) using Welch's periodogram method with a window width of 256s and an overlap of 50%. The spectrum bands were defined as very low frequency 0–0.04 Hz, low frequency 0.04–0.15 Hz, and high frequency 0.15–0.4 Hz.

#### 2.4.2. Arterial Stiffness Index Calculation

**Figure 2:**
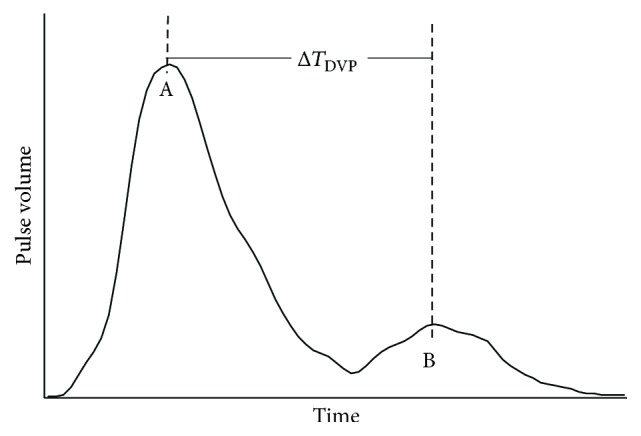
Typical waveform of DVP over a single cardiac cycle. It is derived from a set of real data of the present study. Point A indicates the first peak which is generated by the contraction of left ventricle. Point B is the inflection point which is formed largely by the reflection of pressure from the arteries in the lower extremities back up to the aorta. The time difference between these two points is defined as Δ*T*_DVP_.

DVP waveform was obtained with the pulse oximetry. Three one-minute sections were selected from the 10-minute undisturbed recording both before and after sleep. For each section, a segment of data from 10 consecutive cardiac cycles was extracted and further analyzed by Origin (OriginPro 8 SR0, v8.0724, OriginLab Corporation, Massachusetts, USA). A typical waveform of DVP depicted from a set of real data was displayed in Figure 2. The first peak (point A) and the inflection point (point B) which is defined as the point with the first derivative closest to zero [20] were located by Origin software with manual review. Δ*T*_DVP_ was the time lag between A and B. The mean Δ*T*_DVP_ of the three segments was used to derive ASI based on the equation below, with *h* as the height of the subject:

(1) $$\begin{array}{c} ASI=\frac{h}{\Delta T_{DVP}}. \end{array}$$

#### 2.4.3. Blood Pressure Measurement

Blood pressure was measured before and after sleep with the auscultatory method at the end of the ten-minute supine rest. Measurements were run in duplicate and the mean of the two readings was used for analysis.

#### 2.4.4. Serum Markers Measurement

Blood samples were taken at the end of the ten-minute supine rest after the sleep study. Samples were subjected to a routine laboratory procedure to have the serum separated. ET-1, CRP, IL-6, TNF-*α*, and VEGF were measured with Enzyme-Linked Immunosorbent Assay kits (ET-1, ADI-900-202A, Enzo Life Sciences Inc., New York, USA; CRP, 88-7502, eBioscience, Inc., California, USA; IL-6, BMS213HS, Bender MedSystems GmbH, Vienna, Austria; TNF-*α*, BMS223HS, Bender MedSystems GmbH, Vienna, Austria; VEGF, BMS277/2, Bender MedSystems GmbH, Vienna, Austria). The metabolites of NO were measured by the Griess reaction using prepared kits (ADI-917-020, Enzo Life Sciences Inc., New York, USA) before which samples were ultrafiltered through a 10,000 MWCO filter (Amicon® Ultra-15 10K, Merck Millipore Ltd., County Cork, Ireland) to remove protein as recommended.

### 2.5. Statistical Analysis

Statistical analysis was performed with PASW Statistics 18.0.0. Shapiro-Wilk test was used for the normality test. Normally distributed data are presented as mean (standard deviation) while nonnormally distributed data are presented as median (first quartile, third quartile). Differences were compared by paired *t*-test if the differences between pairs were normally distributed or by Wilcoxon signed-rank test if the differences were not normally distributed. For the measurements of blood pressure, heart rate variability indices, and arterial stiffness index, the overnight change (postsleep measure minus presleep measure) throughout the test night (Δ*T*) was first compared with the change throughout the control night (Δ*C*) to deduct the effect caused by circadian fluctuation. If the difference between Δ*T* and Δ*C* reached significance, we further compared the postsleep measure and the presleep measure under control and test conditions, respectively, to illustrate what changes had occurred. Spearman's rank method was used for correlation analysis. A *P* value less than 0.05 was considered to be statistically significant.

## 3. Results

On the control night, all of the 20 subjects showed no signs of sleep-disordered breathing or other sleep disorders detectable by polysomnography. All subjects proceeded to the second sleep study. During the control study of the eleventh subject, the EEG signal was severely muddied by indeterminate noise for about 100 minutes, leading to the failure of sleep scoring. All the sleep architecture parameters of that night except ArI were not valid for further statistical analysis. The data from the test night of this subject was included in descriptive statistics, but not used for paired comparison.

Table 1 shows the characteristics of the subjects, including the baseline apnea-hypopnea index (AHI) measured on the control nights.

Sleep parameters of both nights are shown in Table 2. The time spent in non-rapid-eye movement (NREM) sleep stage 3 on the test night was significantly less in comparison with that on the control night. The time spent in and the percentage of each of other sleep stages did not differ between the two nights. As expected, the ArI on the test night was significantly higher than that on the control night.

Blood pressure, heart rate variability indices, and arterial stiffness index were measured on four occasions and the results are summarized in Table 3. Compared with the control night, there is a significant overnight elevation in DBP on the test night, but no detectable change in SBP. HRV analysis showed an overnight increase in LF and LHR and a decrease in HF under the test condition. Arterial stiffness index remained relatively stable among the several measurements.

The measures of the serum markers are displayed in Table 4. Repetitive arousals did not elicit any remarkable change in the level of intrinsic vasoactive factors or inflammatory markers of interest.

For the correlation analysis of overnight change in DBP and HRV indices and sleep parameters, the relative difference (overnight change/presleep measure, Δ*T*′) was used instead of the actual overnight difference to minimize the effect introduced by the variation of baseline level. Because LF and HF are linearly correlated with each other and the physiological drive to HF is better understood [23], only HF was used for correlation analysis. As shown in Table 5, HF_Δ*T*′_ was inversely correlated with the time and the percentage of REM sleep, respectively, while LHR_Δ*T*′_ was positively correlated with the same indices. The more REM sleep an individual had, the more the parasympathetic nervous system was inhibited and the more the sympathovagal modulation shifted to sympathetic predomination. The relative overnight changes in DBP, HF, or LHR did not seem to correlate with the amount of sleep of any NREM stage, or ArI.

We further explored the relationship between the relative overnight difference in DBP and that in HF and LHR. The Spearman correlation coefficients are presented in Table 6. The change in DBP was not significantly correlated with alterations in HRV indices.

## 4. Discussion

It has been demonstrated in OSA patients that movement arousals independently predict plasma norepinephrine level [6]. A study in healthy subjects proved the potential cumulative sympathoexcitatory effects of repetitive arousals [7]. Consistent with these previous results, a major finding of the present study showed that repetitive arousals elicited by auditory stimuli led to remarkable change in cardiovascular autonomic control, as evidenced by the overnight increase in LF and LHR and decrease in HF under the test condition. Compared to the aforementioned study which used advanced signal processing to demonstrate the small effect [7], the cumulative effect unveiled in the present study is larger and could be detected by commonly used FFT algorithm. This result suggests a dose-dependent relationship between repetitive arousals and adverse changes in the autonomic status.

Correlation analysis suggested that the shift in sympathovagal balance was associated with the amount of REM sleep. REM sleep is the stage when the SNS is most active, even more so than during wakefulness [24]. However, the effect of REM sleep on the ANS is sleep stage-dependent and the balance shifts towards parasympathetic predomination as one enters NREM sleep [25, 26]. Furthermore, the phenomenon observed in this study is contradictory to what has been observed in OSA patients who typically have reduced REM sleep and higher sympathetic activity before treatment [27, 28]. Whether the statistically significant correlation found in this study is of clinical importance should be investigated in future studies.

ASI measured in the present study did not vary much after the experimental interference, suggesting that the increase in arterial stiffness observed in OSA patients is not an acute phase reaction to repetitive arousals. The two major factors affecting arterial stiffness are the component of vascular wall and the contraction of vascular smooth muscle [19]. It is unlikely for the elastin to collagen ratio in the vascular wall to manifest detectable change within 24 hours. Sympathetic activation could intensify vasoconstriction, yet compared to the effect on muscular arteries, it has less influence on the aorta and large conduit arteries which are the major determinants of ASI [29]. However, it remains unclear whether arteries will stiffen as a consequence of chronic repetitive arousals.

While previous studies have shown that persistent repetitive arousals may lead to endothelial dysfunction due to sleep deprivation and reduction of slow wave sleep [12, 30], there was no acute response in either intrinsic vasoactive factors or inflammatory cytokines to repetitive arousals in the present study. It indicates the influence on endothelium may only become evident after chronic exposure to repetitive arousals. The imbalance in ANS may precede endothelial dysfunction and arterial stiffening as intermediate mechanisms through which repetitive arousals cause adverse cardiovascular outcome.

An important finding of this research is the elevation in DBP from the presleep level as a result of repetitive arousals. On the other hand, no detectable change in SBP was observed. This distinction between the diastolic and systolic blood pressures is consistent with the results from a periodic examination study conducted in Israel [31], which suggests that DBP is the first conventional index to rise in the early course of OSA. Other studies also reported isolated diastolic hypertension as a specific pattern of hypertension associated with sleep-disordered breathing, particularly in the early course of the disease [32, 33]. Hence, DBP might be a potential marker for the early recognition of cardiovascular consequences in OSA patients and its validity is worth further investigation considering its easy acquisition and cost-effectiveness.

A rise in DBP is usually considered to be related to an increase in peripheral resistance [34] which is largely determined by small arterioles while SBP is mainly affected by the conductance vessels [35, 36]. The elevation of DBP in the absence of SBP rising suggests a more pronounced influence of repetitive arousals on peripheral vessels than on the large to moderate vessels. RA-induced increase in peripheral resistance as well as fluctuations in blood pressure are attributed to sympathetic activation [9, 32]. While the changes in SNS activity do not always run in parallel to the changes in blood pressure [7, 37]. The present study did not demonstrate a linear correlation between the change in DBP and the changes in HRV indices and the relationship between ANS activity and BP may be more closely examined using a mathematics model in the future.

## 5. Limitations of the Study

In the present study the first night was regarded as control condition; however this may introduce the “first night effect” into the study. This effect mainly presents as decrease in sleep effectiveness, reduction in REM sleep time, and prolongation of REM sleep latency [38]. This may obscure the difference in the sleep parameters between the two nights with particular influence on REM sleep. However, it has not been reported that “first night effect” would affect cardiovascular measures.

Acoustic stimulation has been commonly and successfully used in both animals [39] and human subjects [4, 7, 9] to induce arousals from sleep. However, this method has its inherent disadvantage in being unable to simulate the mechanism for arousals in OSA. This difference may affect different sleep stages unevenly. In OSA patients, arousals happen more frequently during REM sleep due to the lowest muscular tone during this stage of sleep [40, 41]. But in our research and the preliminary study, we found it harder to induce arousals during REM sleep than during NREM sleep. The stimuli might be incorporated into the ongoing dream during REM sleep rather than producing an awakening [42]. It is not yet clear whether arousals occurring in different sleep stages would incur the same cardiovascular consequences.

All the subjects recruited in the study are all healthy young adults and this may introduce age associated bias in the results. Age-dependent cardiovascular degeneration has been well established and there may be some specific pattern of cardiovascular diseases in elderly people. For example, isolated systolic hypertension is more prevalent in elderly population due to age-related loss of arterial compliance [43]. Whether the findings in the present study also apply to people of other age groups should be studied further. Generally the compensation mechanism is more effective in a younger population. Therefore, we may assume that the cardiovascular response in older people would be more evident.

## 6. Conclusions

In summary, we have demonstrated that repetitive arousals lasting the entire sleep duration could independently cause adverse cardiovascular consequences. More specifically, the experimental arousals increase sympathetic activity and decrease parasympathetic activity, leading to a shift of the sympathovagal modulation towards the sympathetic predomination. The cumulative effects on cardiovascular autonomic control seem to be associated with the amount of REM sleep. Exposure to the repetitive arousals also causes an elevation in postsleep DBP from presleep level but does not affect SBP significantly. Disturbance to ANS may occur prior to endothelial dysfunction and increase of arterial stiffness as an acute reaction to repetitive arousals.

## Figures and Tables

**Table 1:** Characteristics of the subjects.

Age/y	25.0 (2.1)
Height/m	1.76 (0.05)
Weight/kg	71 (7)
BMI/kg·m^−2^	22.9 (1.9)
Sleep duration/h	7.0 (6.4, 7.0)
Baseline AHI/h^−1^	0.30 (0.02, 0.78)

BMI, body mass index; AHI, apnea-hypopnea index.

**Table 2:** Sleep parameters on the control night and the test night.

	Control	Test
TST/min	390 (44)	368 (54)
N1/min	42 (16, 46)	39 (17)
N2/min	263 (52)	248 (40)
N3/min	21 (17)	4 (0, 16)^*∗*^
REM/min	66 (22)	70 (22)
N1%	11.2 (4.0, 12.2)	10.7 (4.3)
N2%	67.1 (8.7)	68.1 (7.6)
N3%	5.2 (4.2)	1.3 (0.0, 4.1)
REM%	17.0 (5.6)	18.4 (4.4)
ArI/h^−1^	15.1 (3.1)	43.9 (9.1)^†^

TST, total sleep time; N1, non-rapid-eye movement sleep stage 1; N2, non-rapid-eye movement sleep stage 2; N3, non-rapid-eye movement sleep stage 3; REM, rapid-eye movement sleep; N1%, the percentage of N1; N2%, the percentage of N2; N3%, the percentage of N3; REM%, the percentage of REM; ArI, arousal index; ^*∗*^*P* < 0.05 from control condition; ^†^*P* < 0.001 from control condition.

**Table 3:** Measurements of blood pressure, heart rate variability indices, and stiffness index.

	Control		Test	
	Presleep	Postsleep	Presleep	Postsleep
SBP/mmHg	118 (6)	116 (6)	115 (6)	115 (7)
DBP/mmHg^△^	80 (6)	79 (8)	80 (5)	83 (5)^*∗*^
LF/n.u.^▲^	54 (16)	59 (13)	46 (17)	72 (12)^ †^
HF/n.u.^▼^	46 (16)	41 (13)	54 (17)	28 (12)^ ‡^
LHR^▲^	1.2 (0.8, 1.8)	1.4 (1.1, 2.1)	0.7 (0.4, 1.4)	2.7 (1.8, 5.0)^ †^
ASI/m·s^−1^	5.50 (0.44)	5.55 (0.31)	5.60 (0.43)	5.50 (0.45)

SBP, systolic blood pressure; DBP, diastolic blood pressure; LF, low frequency; HF, high frequency; n.u., normalized units; LHR, low frequency to high frequency ratio; ASI, arterial stiffness index. ^△^The overnight change (postsleep – presleep) under the test condition is higher from that under the control condition with *P* < 0.05. ^▲^The overnight change under the test condition is higher from the control condition with *P* < 0.001. ^▼^The overnight change under the test condition decreases from the control condition with *P* < 0.001. ^*∗*^Postsleep measurement is increased from the presleep level with *P* < 0.05. ^†^Postsleep measurement increases from the presleep level with *P* < 0.001. ^‡^Postsleep measurement is decreased from the presleep level with *P* < 0.001.

**Table 4:** Measurements of serum markers.

	Control	Test	*P*
NO/*μ*mol·L^−1^	46 (26)	46 (18)	N/S
ET-1/pg·ml^−1^	1.58 (1.27, 2.68)	1.59 (1.04, 2.61)	N/S
IL-6/pg·ml^−1^	0.63 (0.41, 0.88)	0.69 (0.46, 1.01)	N/S
CRP/pg·ml^−1^	8.8 (4.8, 18.9)	6.6 (4.6, 18.1)	N/S
TNF-*α*/pg·ml^−1^	0.86 (0.82, 0.96)	0.88 (0.84, 0.94)	N/S
VEGF/ng·ml^−1^	0.15 (0.09, 0.23)	0.14 (0.09, 0.20)	N/S

NO, nitric oxide; ET-1, endothelin-1; IL-6, interleukin-6; CRP, C-reactive protein; TNF-*α*, tumor necrosis factor-*α*; VEGF, vascular endothelial growth factor; N/S, not significant.

**Table 5:** Correlation analysis of HF_Δ*T*′_, LHR_Δ*T*′_, and DBP_Δ*T*′_ with sleep parameters.

	DBP_Δ*T*′_	HF_Δ*T*′_	LHR_Δ*T*′_
TST	−0.006	−0.439	0.424
N1	0.125	−0.131	−0.169
N2	−0.034	−0.133	0.188
N3	0.251	−0.120	0.112
REM	−0.127	−0.656^*∗*^	0.662^*∗*^
N1%	0.167	0.269	−0.326
N2%	−0.199	0.135	−0.119
N3%	0.235	−0.101	0.094
REM%	−0.128	−0.602^*∗*^	0.624^*∗*^
ArI	0.134	0.061	−0.108

Δ*T*′, relative overnight difference (overnight change/presleep measure) on the test night; DBP, diastolic blood pressure; HF, high frequency power; LHR, low frequency to high frequency ratio; TST, total sleep time; N1, non-rapid-eye movement sleep stage 1; N2, non-rapid-eye movement sleep stage 2; N3, non-rapid-eye movement sleep stage 3; REM, rapid eye movement sleep; N1%, the percentage of N1; N2%, the percentage of N2; N3%, the percentage of N3; REM%, the percentage of REM; ArI, arousal index. ^*∗*^The correlation is significant with *P* < 0.05.

**Table 6:** Correlation between DBP_Δ*T*′_ and Δ*T*′ in HRV variables.

	DBP_Δ*T*′_	
	*r*	*P*
HF_Δ*T*′_	0.096	N/S
LHR_Δ*T*′_	−0.175	N/S

Δ*T*′, relative overnight difference (overnight change/presleep measure) on the test night; DBP, diastolic blood pressure; HF, high frequency power; LHR, low frequency to high frequency ratio; N/S, not significant.
